# The development and pilot testing of the OroFacial Awakening Symptoms Questionnaire (OFASQ)

**DOI:** 10.22514/jofph.2025.013

**Published:** 2025-03-12

**Authors:** Ovidiu Ionut Saracutu, Alessandro Bracci, Matteo Val, Anna Colonna, Marco Ferrari, Daniele Manfredini

**Affiliations:** ^1^Department of Medical Biotechnologies, School of Dentistry, University of Siena, 53100 Siena, Italy; ^2^Department of Neurosciences, School of Dentistry, University of Padova, 35128 Padova, Italy

**Keywords:** STAB, BruxScreen, Orofacial awakening symptoms, Bruxism, Sleep bruxism, TMD, NRS-11 scale, Face validity, Pilot testing

## Abstract

**Background**: The evolution of concepts that have featured the last decade in the field of bruxism led to the necessity of providing clinicians and researchers with adequate tools for the assessment of bruxism, such as the Standardized Tool for the Assessment of Bruxism (STAB) and the BruxScreen. The former is a multidimensional evaluation tool for the evaluation of bruxism status, while the latter is an instrument that could potentially find its applicability in large-scale epidemiological research projects for screening purposes. However, both tools lack the evaluation of orofacial symptoms at awakening, which can be predictive of temporomandibular disorders (TMDs) pain intensity and prognosis. The aim of this paper is to discuss the development of a novel tool, the OroFacial Awakening Symptoms Questionnaire (OFASQ). This questionnaire could be integrated into the STAB to investigate the presence of orofacial symptoms upon awakening and enhance knowledge of the relationship between sleep-time bruxism activities and potential clinical consequences. The OFASQ consists of a preliminary screening question about the presence or absence of a series of orofacial symptoms upon awakening and five items that evaluate the amount of pain and impairment they cause. **Methods**: For pilot testing, the OFASQ was administered to a diverse group of 85 subjects, including dental practitioners of various specialties, postgraduate and undergraduate dentistry students and patients. **Results**: Following the face validity and pilot testing phase, it emerged that OFASQ could represent a valid tool for quantifying the intensity and severity of orofacial symptoms upon awakening in everyday clinical practice. **Conclusions**: The OFASQ tool is considered ready for more in-depth clinical testing. The authors do not exclude the possibility of minor editing to the tool following further, more in-depth tests.

## 1. Introduction

In the last ten years, the construct of bruxism and its assessment strategies have been completely revisited. In 2013, a panel of experts in the field defined bruxism as a “repetitive jaw-muscle activity characterized by clenching or grinding of the teeth and/or by bracing or thrusting of the mandible”, which can have two different circadian manifestations during sleep and wakefulness [1]. In the following years, the need to elucidate some aspects of the definition emerged [2], and in 2018, a second consensus paper provided two separate definitions based on the circadian rhythm. Sleep bruxism (SB) was defined as a “masticatory muscle activity during sleep that is characterized as rhythmic (phasic) or non-rhythmic (tonic) and is not a movement disorder or a sleep disorder in otherwise healthy individuals”, while awake bruxism (AB) was defined as a “masticatory muscle activity during wakefulness that is characterized by repetitive or sustained tooth contact and/or by bracing or thrusting of the mandible and is not a movement disorder in otherwise healthy individuals” [3]. Given the potentially equivocal interpretation of the terms “otherwise healthy individuals”, five years later, some of the authors wrote an explanatory note to clarify that in no circumstances could bruxism be seen as “the” disorder, being rather a sign of an underlying or associated condition [4]. As such, bruxism activities may be seen as a risk factor for certain clinical consequences (*e.g.*, pathological tooth wear, myofascial pain, difficulties in mouth opening, temporomandibular joint pain and teeth soreness) [5, 6].

Despite the reconceptualization of bruxism as a masticatory muscle activity, a critical gap in knowledge was still present regarding its assessment. The 2013 grading system, introduced in the first consensus paper, was a tentative proposal that has never been clinically validated for the different types of bruxism [5]. Thus, following up the need to provide clinicians and researchers with a standardized and universally accepted instrument for bruxism evaluation, an enlarged group of experts with respect to the authors of the consensus definitions paved the way for the creation of a multidimensional Standardized Tool for the Assessment of Bruxism (STAB) [7], which was published in 2024 after five years of debates and workshops [8]. The STAB consists of an Axis A assessing bruxism based on the different methods available, the subject-based (self-report), the clinically based (clinical examination), and instrumentally based (*e.g.*, Ecological Momentary Assessment (EMA), wake-time and night-time electromyography and polysomnography) assessment, together with bruxism consequences, and an Axis B evaluating risk factors and co-occurring underlying conditions (*e.g.*, anxiety and depression, gastroesophageal reflux disease, and orofacial motor disorders). The comprehensiveness of the STAB makes it a tool that might not be applicable in all integrity into everyday practice by clinicians and researchers, who should instead select and pick up from the STAB toolkit the items/domains that are primarily suitable for their investigation [8]. Thus, in parallel to the STAB, a screening instrument (*i.e.*, BruxScreen) was proposed by a working group led by the chairmen of the bruxism panel [9] as an easier tool to apply for large-scale epidemiological studies and meet the proposed A4 principle [3]. The BruxScreen is composed of two parts. The BruxScreen-Q (items to be answered by the patient) is based on a series of questions regarding the frequency of bruxism and jaw symptoms. The BruxScreen-C (items to be assessed by the clinician) is instead based on a series of clinical findings already proposed in the STAB [9].

In both the STAB and the BruxScreen, the frequency of bruxism behaviors and jaw symptoms at awakening is assessed using a Likert-type scale [8, 9, 10]. Compared to dichotomous answers, such a scale provides a quantitative esteem of the frequency of such symptoms. However, neither tool quantifies symptoms intensity, such as pain, functional limitation, difficulty opening the mouth and dental soreness upon awakening, which might be necessary for performing clinical and research investigations to enhance knowledge on the relationship between sleep-time bruxism activities and clinical consequences and outcomes. Indeed, the adoption of the OFASQ could potentially unveal a causal relationship, according to the Bradford Hill criteria [11], between a series of conditions that put a strain on the masticatory system, such as clenching and grinding of the teeth during night [12], with the orofacial symptoms present at awakening. A large epidemiological study conducted by Velly *et al*. [13] on a sample of 1901 Americans with painful temporomandibular disorders (TMD) revealed that on one hand 82% experienced jaw pain or stiffness upon awakening, and on the other hand, in almost 75% of them the pain attributed to TMD had been present for more than 6 months. Within the study population, 40.3% presented low-intensity pain on the graded chronic pain scale (GCPS) (GCPS grade I), while 27.7% experienced high-intensity pain (GCPS grade IIa). In addition, a recent cohort study published by the same author in 2022 suggested that pain reported upon awakening might be a predictors of long-lasting TMD pain, with an odds ratio of 1.66 [14]. However, when analyses included the potential risk factors and confounders, the significant odds ration did not remain. Nevertheless, in orofacial pain patients, pain at awakening significantly correlated with pain intensity and muscle tenderness during the rest of the day [15]. These findings suggest that awakening symptoms can provide clinicians with potentially useful information concerning the whole clinical scenario. Therefore, it could be beneficial to complement the bruxism evaluation with a questionnaire evaluating patients’ intensity of symptoms upon awakening.

Based on the above, this paper reports the development of a novel tool, the OroFacial Awakening Symptoms Questionnaire (OFASQ). This questionnaire is designed to assess the severity of orofacial symptoms upon awakening in terms of pain intensity and interference with everyday activities. The paper also details the pilot testing phase and face validity assessment of the OFASQ. The study hypothesis is that, following the pilot testing phase, the OFASQ will demonstrate a satisfactory level of face validity. This means that an informal review of the questionnaire by non-experts will confirm its clarity, comprehensibility, and appropriateness for measuring the investigated outcome [16]. Once this initial phase is complete, the questionnaire will be ready for more in-depth clinical testing.

## 2. Methods

### 2.1 Conceptualization and creation of the questionnaire

Considering that no other questionnaire specifically related to the symptoms present upon awakening is available in the literature, the authors had to conceptualize the OFASQ according to their expertise in the field and their knowledge regarding other existing questionnaires. The OFASQ was ideated by three authors of the paper (OIS, AC, AB) as part of the PhD project of the leading author. Each author independently created a draft version. An online meeting was then organized to compare the three proposed versions and discuss the discrepancies concerning the included items. After the discussion, a single version of the OFASQ was prepared. One of the authors (DM) then reviewed the first draft of the OFASQ, which was then emailed to the other co-authors. After several rounds of email exchanges, an in-person meeting was organized to let each author free to propose any further suggestions before reaching a final consensus on the definitive version of the OFASQ. The English language was adopted for all these phases, and a certified mother tongue language specialist was involved in checking and refining grammar and syntax. This version, described below, was then subjected to pilot testing to evaluate the face validity assessment in terms of the questions’ comprehensibility and the administration’s feasibility.

The pilot testing of the OFASQ was performed at the University of Siena, Dental School clinic, Italy, following the procedure proposed by de Vet *et al*. [16] and similarly to the strategy that was adopted by Lobbezoo *et al*. [9] for the BruxScreen. On 08 April 2024, the proposed version of the OFASQ was administered to a diverse sample of 85 individuals. This sample included 40 international undergraduate dentistry and dental prosthodontics students (12 males, 28 females, age range 20–25 years), 15 international postgraduate orofacial pain students (3 males, 12 females, age range 27–54 years), 10 dentists (8 males, 2 females, 4 prosthodontists, 3 endodontists, 1 oral pathologist, 2 general dentists, age range 49–63 years) and 20 patients (10 males, and 10 females, age range 43–81 years). An Italian version was used for non-English participants, who were mostly patients, following the established indications for forward and back translation [17].

To assess the face validity of the OFASQ, authors employed a range of rigorous strategies. These included the think-aloud technique [18], which encouraged participants to verbalize their thoughts as they completed the questionnaire, as well as observations and retrospective verbal probing [16]. The latter method allowed examiners to question the participants about their understanding of the content and the interpretation of each item, providing valuable insights into the questionnaire’s comprehensibility and relevance.

For this type of study, according to the local legislation, approval from the School of Dentistry Ethical Advisory Board was achieved (#0127-2024).

### 2.2 Description of the self-report questionnaire

The questionnaire contains a first preliminary question with a dichotomous answer (Yes/No) to assess whether the patient has ever experienced any orofacial symptoms on awakening in the last month, such as difficulties to open the mouth, stiffness, tightness, pain in the jaw muscle, temples, or temporomandibular joint. If the answer is positive, the patient must answer five additional questions about the orofacial symptoms.

The first question concerns the difficulty experienced by the patient in opening the mouth upon awakening. The item was taken from the Fonseca questionnaire [19], modifying the answer from a dichotomic option (Yes/No) to the numerical rating scale-11 (NRS-11), proposed for the first time by Downie in 1978 [20]. In such a scale, patients are requested to select among 11 possible answers, ranging from 0 (no interference) to 10 (extreme change), one which represents the level of impairment the symptoms cause. Several other scales for grading pain have been proposed in the literature [21], however, the NRS-11 scale results to be the most accurate in reproducing the pain intensity [22]. Such scale was adopted for all the items present in the questionnaire to obtain a quantitative perception of the patient and assess the severity of the impairment and pain, which is missing in the STAB and the BruxScreen. The second and third questions are based on the first two items of the TMD Pain Screener [23], with a modification related to the answer option. Question 2 relates to the amount of stiffness, fatigue or tightness in the jaw muscle or the temple on awakening. The response is given based on a NRS-11 scale. Question 3 assesses the presence of pain in the jaw muscle and temple on awakening. The question is divided into two parts, 3a and 3b. Item 3a reports the intensity of pain, while item 3b investigates the level of interference of the symptom on daily activity on a NRS-11 scale. In addition, a fourth question, also divided into two parts was added to gather information about temporomandibular joint (TMJ) pain upon awakening. Item 4a assesses the intensity of pain in the ear, or in front of the ear, or on both sides. Question 4b measures the amount of interference this would have in daily activities. The question was taken from the first item of the diagnostic criteria for temporomandibular disorders (DC/TMD) pain section of the symptom questionnaire [24]. Given the impact of TMD pain on the quality of life [25, 26, 27], question 4b was considered necessary to gather information on how debilitating the symptoms can be. However, for the sake of brevity, it was decided not to add a question related to each type of functional activity that can be impaired by TMJ pain. For both questions 4a and 4b, the use of the NRS-11 scale instead of the dichotomous option was considered necessary to correlate the pain level with other potential clinical variables. Finally, a fifth question was added to assess the possible level of discomfort related to teeth soreness the patient can experience at awakening, specifying that this type of pain is different compared to the pain of pulpal origin. Headache can also be a symptom related to sleep bruxism, which clinicians should not underestimate. However, items on it are already present in the STAB, thus, we did not consider necessary to implement in the OFASQ, specific questions on headache, which can be better investigated by means of their questionnaires present in the literature, such as the Headache-Attributed Restriction, Disability, Social Handicap and Impaired Participation (HARDSHIP) questionnaire [28].

## 3. Results

### 3.1 Pilot testing

During the questionnaire administration, the examiners evaluated the perceived content and interpretation of the items. In the first instance, thanks to the think-aloud protocol [18], the authors took notes of the observations made by the participants during the filling of the OFASQ. The examiner adopted the “probing” method to assess participants’ impressions and inquiries about the questionnaire [16]. The pilot testing led to the following considerations:

• Comprehensibility: 18 of the 20 patients involved in the pilot testing reported a high comprehension of the questionnaire, the items included, and the NRS-11 scale. One patient reported that he would appreciate a better explanation of the source of tooth soreness upon awakening. Another patient reported that she could not understand the different localization between jaw muscle pain and temporomandibular joint pain. All the dentists and most students reported a good comprehension of all the items. Three undergraduate dentistry students reported that they would have preferred that the possible causes of reduced mouth opening upon awakening had been described.

• Feasibility: All the participating patients, students, and dentists found the questionnaire straightforward and easy to fill out. The fact that it took them between one and five minutes to complete it further underscores the user-friendly nature of the tool. Importantly, all the dentists agreed that the questionnaire could be a simple and cost-effective tool to administer in a clinical dental setting.

• Miscellaneous: One patient could not understand where the temples are located and needed additional verbal explanation. Two students did not know what is the level of pain on the NRS-11 scale that would make it clinically relevant.

Based on the outcome of the pilot testing and following the discussion among the paper’s authors, it was decided to add a specification regarding tooth soreness. Since some of the patients were unaware of the existence of the nociceptive capabilities of the dental pulp tissue, it was decided to specify that toothache is caused by caries, a term well-known by the general population. Moreover, being pain an unpleasant sensory and emotional experience [29] and a subjective perception, it was decided that the setting of a specific threshold point could be too arbitrary, leading to misinterpretation of the patient’s pain status.

### 3.2 Face validity assessment

The face validity of the OFASQ, *i.e.*, the degree to which the tool can measure the outcome of interest, was assessed by the authors after collecting feedback from the participants who took part in the pilot study. However, as also discussed by Lobbezoo *et al*. [9], the outcome cannot be measured due to the absence of a reference standard concerning face validity per se [16]. Despite this, the authors of the paper concluded that OFASQ would likely represent a valid tool for the assessment to quantify the intensity and severity of orofacial symptoms upon awakening (*i.e.*, difficulty in opening the mouth, stiffness, fatigue, tightness and pain in the jaw muscles or temples, pain at the level of the temporomandibular joint, teeth soreness) in everyday clinical practice. Moreover, the short time required for its compilation makes it an easy tool to administer in dental settings.

## 4. Discussion

This paper aims to describe the development of a screening tool for assessing orofacial symptoms upon awakening, evaluating their intensity and the interference they have in everyday activities. The tool consists of a questionnaire with five items to be answered by patients using a graded scale based on continuous, ordinal variables. The first version of the OFASQ proposed in this paper was pilot-tested in a sample of 85 participants composed of patients, dentists, and students of undergraduate and master dentistry courses. For this purpose, the authors adopted the procedures de Vet *et al*. [16] proposed in the textbook “Measurement in Medicine”. The pilot test revealed the necessity of adding to question 5 a specification on the fact that dental caries do not cause the type of pain investigated. The face validity process demonstrated that the tool could be a valid instrument to assess the orofacial symptoms at awakening in a feasible way. The definitive version of the OFASQ is shown in **Supplementary material**. The face validity of the OFASQ marks the first step in its validation process, making it a tool ready for testing in clinical settings. However, its criterion validity, reliability, responsiveness to change, and interpretability are yet to be established. Further tests are needed to verify if the OFASQ meets the abovementioned criteria [16]. The authors are fully committed to refining the tool based on any criticism that may emerge from in-depth clinical testing of the questionnaire. This dedication to refining the tool underscores its potential to significantly impact patient research and care as a relevant complement to bruxism evaluation instruments.

The rationale behind the questionnaire is that, even though the STAB and BruxScreen contain items related to symptoms upon awakening, they lack the evaluation of their intensity and limit their analysis to the frequency or the simple presence/absence. On the other hand, orofacial pain at awakening is a relatively common finding in TMD patients [13], and its severity could be a predictor of the prognosis [14] and intensity [15]. Thus, the clinician could gather fundamental information by evaluating its intensity and impact.

The decision to adopt an NRS-11 is justified by the fact that this scale can provide a good evaluation of pain with high sensitivity and good capability for data analysis [22]. The proposed questionnaire was used to evaluate the level of impairment during everyday life activity caused by awakening orofacial symptoms. Given the short-term natural fluctuating of TMD symptoms and treatment needs [30], the authors considered it necessary to investigate the presence of orofacial symptoms upon awakening in the last 30 days. For the same reason, in the DC/TMD [24], the use of the second version of the Graded Chronic Pain Scale was proposed instead of the conventional six-month span. The 30-day version of the scale was validated in a large group of 521 TMD patients [31]. Moreover, the GCPS 2.0 proved to have the same accuracy as the GCPS 1.0 in screening TMD pain and dividing them into different subtypes [32].

Considering the lack of orofacial symptoms quantification present upon awakening in the STAB and the Bruxscreen and their importance due to the predictive values they can have on TMD pain [15], the OFASQ can serve as a tool to better investigate their potential causes, such as bruxism activities occurring during sleep time [33]. Many studies have found an association between sleep bruxism and TMD pain [34, 35, 36, 37, 38], especially myogenous pain [37], but no research article in the literature have investigated the intensity of the orofacial symptoms at awakening and the frequency of oral behaviors during sleep-time. Thus, the OFASQ could represent an intriguing integration to the STAB, which already contains all the possible items for sleep bruxism assessment.

Within these premises, it is important to consider that pain has been defined as “an unpleasant sensory and emotional experience associated with, or resembling that associated with, actual or potential tissue damage”, which implies that it is a subjective occurrence, not necessarily related to the degree of tissue damage [27]. The inter-individual difference in experiencing pain also has profound implications for the patient’s treatment response. Studies performed on healthy volunteers have shown that, as a response to the same pain-inducing stimulus, the intensity reported on the NRS-11 scale ranges from slightly above one to slightly below nine [39]. A possible explanation for this finding is that several factors contribute to the interindividual heterogeneous response to painful stimuli. In the first instance, genetic factors play an important role [40], as well as gender [41, 42, 43]. Interestingly, gender of the examiner was also found to have a significant impact on the report of the pain [44]. In addition, psychological factors have profound implications for pain perception [45]. The biopsychosocial model, which is currently adopted in the field of TMD, is the one that better explains the complex interrelationship between psychological distress and pain sensitivity [46, 47]. Under this scenario, laboratory studies have shown that pain catastrophizing is the factor that plays an important role in increasing pain sensitivity but also suppressing the physiological mechanism of diminishing pain perception [48]. Clinical studies performed on TMD patients in dental settings have revealed the impact of the psyche on the intensity of the pain [49, 50].

The OFASQ should be viewed as an instrument to assess how the patient experiences orofacial symptoms at awakening, *i.e.*, how the central nervous system interprets the painful stimuli [42], and to possibly link the symptoms to the various bruxism activities [51, 52, 53, 54].

The main limitation of the study design is that only the face validity of the questionnaire was assessed, whilst any data on its sensitivity and specificity could be gathered. Face validity, while being the first necessary fundamental component of a questionnaire, is considered one of the weakest forms of validity [55]. Thus, the OFASQ, in its current state, cannot be viewed as a tool that is ready for definitive use in the clinical setting yet, as further studies are needed to measure its validity and reliability. In particular, future investigations should evaluate the criterion validity by measuring the correlation between the OFASQ and similar items taken from validated questionnaires. Moreover, in addition to calculating Cronbach’s alpha coefficient, the test-retest reliability should be assessed on a sample of patients by administering the questionnaire twice to the same participants and measuring the correlation between the results [55].

## 5. Conclusions

Following the positive results of pilot testing and the face validity of the OFASQ, a feasible and comprehensible tool for evaluating orofacial symptoms at awakening has been developed. The tool is considered valid for further testing to assess its criterion validity, reliability, and responsiveness to change. The authors do not exclude the possibility of adding minor editing to the tool, following further, more in-depth tests.

## Supplementary Material

Supplementary material associated with this article can be found, in the online version, at https://files.jofph.com/ files/article/1899711951495151616/attachment/ Supplementary%20material.docx.


