# Supplementary material for: The development and pilot testing of the OroFacial Awakening Symptoms Questionnaire (OFASQ)
Source: J Oral Facial Pain Headache. 2025 Mar 12;39(1):134–40. doi: 10.22514/jofph.2025.013 (PMC11934730; doi:10.22514/jofph.2025.013)
Supplement: Supplementary file 1 [file Supplementary-material.docx]

Supplementary material

OroFacial awakening symptoms questionnaire OFASQ

In the last 30 days, did you experience any orofacial symptoms on awakening such as difficulties to open your mouth, or stiffness, tightness, or pain in your jaw muscles, temples or temporomandibular joint?

Yes

No

If yes, please fill out the following questions:

1 - In the last 30 days, have you had any difficulties to open your mouth on awakening?

Please rate how much has this symptom interfered in your daily activities, where 0 is “no interference” and 10 is “extreme change”.

No interference 0 1 2 3 4 5 6 7 8 9 10 Extreme change

2 - In the last 30 days, have you had any stiffness, fatigue or tightness in your jaw muscles or in your temples on awakening?

Please rate how much has this symptom interfered in your daily activities, where 0 is “no interference” and 10 is “extreme change”.

No interference 0 1 2 3 4 5 6 7 8 9 10 Extreme change

3 - In the last 30 days, have you had any pain in your jaw muscles or in your temple on awakening?

a. Please rate how intense was this symptom on average, where 0 is “no pain” and 10 is “pain as bad as could be”.

No pain 0 1 2 3 4 5 6 7 8 9 10 Extreme pain

b. Please rate much has this symptom interfered in your daily activities, where 0 is “no interference” and 10 is “extreme change”.

No interference 0 1 2 3 4 5 6 7 8 9 10 Extreme change

4 - In the last 30 days, have you had any pain in your temporomandibular joint (TMJ) on awakening? (TMJ is the joint near the ear that allows you to open and close the mouth).

a. Please rate how intense was this symptom on average, where “0” is no pain and “10” is pain as bad as could be.

No pain 0 1 2 3 4 5 6 7 8 9 10 Extreme pain

b. Please rate much has this symptom interfered in your daily activities, where 0 is “no interference” and 10 is “extreme change”.

No interference 0 1 2 3 4 5 6 7 8 9 10 Extreme change

5 - In the last 30 days, have you had teeth soreness on awakening?

(Do not confuse it with any toothache caused by caries). Please rate how intense was this symptom on average, where “0” is no pain and “10” is pain as bad as could be.

No pain 0 1 2 3 4 5 6 7 8 9 10 Extreme pain
